# Generation of a monoclonal antibody recognizing the CEACAM glycan structure and inhibiting adhesion using cancer tissue-originated spheroid as an antigen

**DOI:** 10.1038/srep24823

**Published:** 2016-04-21

**Authors:** Yumi Sato, Hiroaki Tateno, Jun Adachi, Hiroaki Okuyama, Hiroko Endo, Takeshi Tomonaga, Masahiro Inoue

**Affiliations:** 1 Department of Biochemistry Osaka Medical Center for Cancer and Cardiovascular Diseases, 1-3-3 Nakamichi, Higashinari-ku, Osaka 537–8511, Japan; 2 Division of Molecular Virology and Oncology, Cancer Research Institute, Kanazawa University, Kakuma-machi, Kanazawa 920-1192, Japan; 3 Biotechnology Research Institute for Drug Discovery (BRD), National Institute of Advanced Industrial Science and Technology (AIST), 1-1-1 Umezono, Tsukuba, Ibaraki 305-8568, Japan; 4 Laboratory of Proteome Research, National Institute of Biomedical Innovation, Health and Nutrition. 7-6-8 Saito-Asagi, Ibaraki-shi, Osaka 567-0085, Japan

## Abstract

Spheroids cultured directly from tumours can better reflect *in vivo* tumour characteristics than two-dimensional monolayer culture or three-dimensional culture of established cell lines. In this study, we generated antibodies by directly immunizing mice with primary-cultured living spheroids from human colorectal cancer. We performed phenotypic screening via recognition of the surface of the spheroids and inhibition of their adhesion to extracellular matrices to identify a monoclonal antibody, clone 5G2. The antibody inhibited cell migration in two-dimensional culture and promoted cell detachment. Western blotting and immunohistochemistry detected the 5G2 signal in many colorectal cancer spheroids, as well as patient tumours, but failed to detect in various cell lines examined. We found that 5G2 recognized the Le^a^ and Le^c^ on N-glycan, and their major carrier proteins were CEACAM5 and CEACAM6. Pre-incubation of the spheroids with 5G2 impaired translocation of integrin β4 from the lateral membrane to the contact interface between the extracellular matrix when embedded in it. As we successfully obtained a functional antibody, which antigen was glycan structures and lost in cell lines, cancer tissue-originated spheroids can be a useful antigen for generating novel anti-cancer antibodies.

Three-dimensional (3D) cell culture systems are rapidly developing^1^,^2^ because they reflect *in vivo* characteristics better than conventional two-dimensional (2D) monolayer culture in regards to architecture, drug sensitivity^3^,^4^, gene expression, protein characteristics, and signal transduction^5^,^6^,^7^. For example, HER2 changes its dimerization partner from HER3 in 2D culture to HER2 in 3D culture, resulting in sensitivity to trastuzumab and intracellular signalling pathways^8^. On the other hand, primary culture systems are a more promising approach than established cell lines. A glioma study indicated that genomic profiles are frequently altered from those of parental tumours during short-term 2D culture, but they are more stable and representative of parent tumours when cultured in 3D spheroids^9^. Therefore, spheroids cultured directly from tumours can best reflect *in vivo* tumour characteristics.

We previously established an efficient method for primary spheroid culture from patient tumours, the cancer tissue-originated spheroid (CTOS) method^10^. The principle of the CTOS method is to maintain cell-cell contact throughout the preparation and culture process. CTOS-derived xenotumours preserve the features of the patient tumours from which they originate. These characteristics of CTOSs promise more reproducible studies of patient tumours than cell lines. Loss of cell polarity is considered a hallmark of cancer^11^,^12^,^13^, but adenocarcinomas form gland-like structures, indicating that cell polarity is not completely lost, but retained to some extent in most cases. CTOSs from differentiated colorectal cancer (CRC) maintain their original differentiation status in CTOS-derived xenotumours^10^. In colorectal CTOSs, the apical membrane forms on the spheroid surface under floating culture conditions (i.e., suspension culture), but it forms on the surface of the lumens inside gel-embedded CTOSs (Okuyama, Am J Pathol, in press).

Circulating tumour cells (CTCs) are regarded as an origin of cancer metastasis, and the presence of clusters of CTCs reportedly correlates with metastatic potential and worse prognosis than single CTCs^14^,^15^,^16^. In the region of microvessel invasion in CRC tumours, we found that some of the cancer cell clusters have the apical membrane on their surface (Okuyama, Am J Pathol, in press), which is quite similar to the CTOSs cultured under floating conditions. Thus, the adhesion of apical membrane protein can be a critical event for cell clusters in metastasis.

Phenotypic screening of new drug candidates is based on their effectiveness against phenotypes of the target disease. In contrast, targeted-based screening is based on the target molecule and the molecular mechanism of action for effective drugs. Phenotypic screening is more successful than targeted screening for first-in-class drugs^17^. As a large number of novel anti-cancer drugs are monoclonal antibodies, the generation of monoclonal antibodies using phenotypic screening is a promising approach^18^. Some studies have reported phenotypic screening for anti-cancer antibodies using phage display^19^,^20^,^21^ or hybridomas^22^, though they used cell lines or 2D cultured patient-derived cells.

In the present study, we aimed to generate antibodies by phenotypic screening using CTOSs, as colorectal CTOSs, which preserve the architecture of the *in vivo* tumour, may reproduce the protein localization and modification. We selected the hybridoma approach because high affinity antibodies can be expected^23^. We directly immunized mice with the CTOSs and obtained monoclonal antibodies that recognized molecules on the apical membrane, further screening them based on their inhibition of adhesion. We obtained a monoclonal antibody, 5G2, and the antigen was detected in various CTOSs, as well as original tumours, but not cell lines. We demonstrate that 5G2 recognizes the glycan structure of CEACAM5 and CEACAM6, and investigated the mechanisms underlying adhesion inhibition.

## Results

### Generation of monoclonal antibodies against the CTOS surface

**Figure 1:**
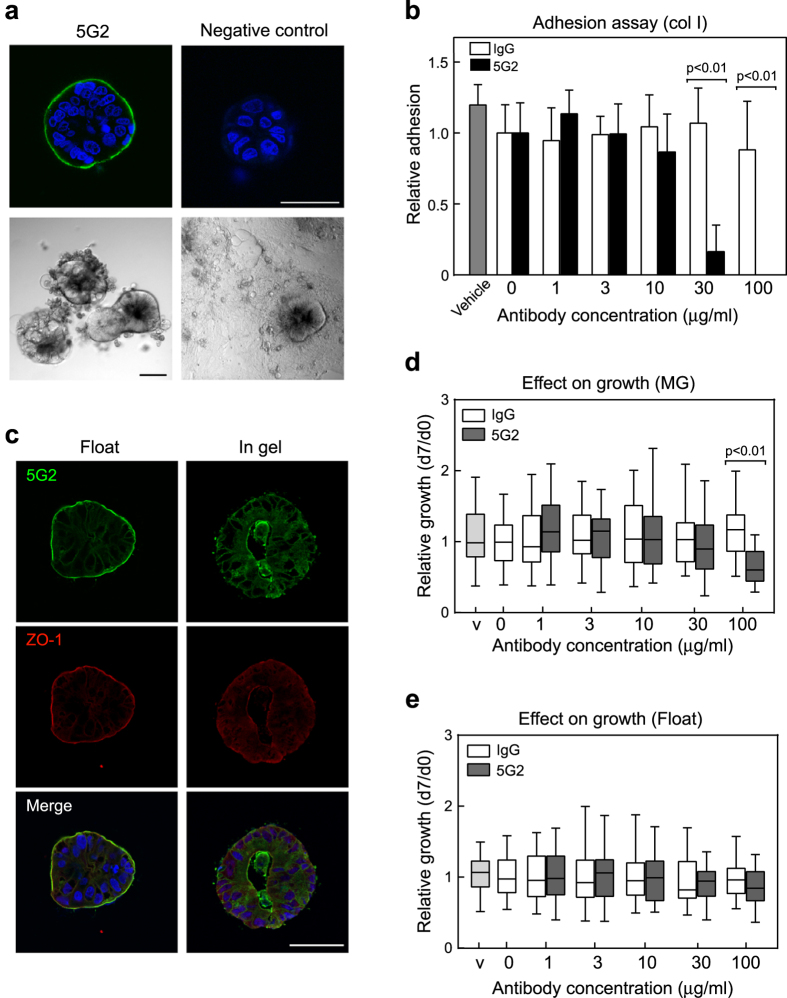
Generation of monoclonal antibody against the CTOS surface. (**a**) Top, whole mount immunocytochemistry (ICC) without permeabilization of live CTOSs with 5G2 mAb. CTOSs were incubated with 1 μg/ml purified 5G2 mAb or mouse IgG3κ isotype control and observed by confocal microscopy. Scale bar, 50 μm. Bottom, phase contrast images of an adhesion assay. CTOSs were cultured for 5 days with or without 5G2 hybridoma culture media on a type I collagen-coated dish. Scale bar, 100 μm. (**b)** Quantification of adhesion to type I collagen-coated plates based on the percentage of adhered CTOSs. Data indicate mean ± SD from three independent experiments. Each condition was performed in triplicate and data are shown relative to the negative control (non-treated) in each experiment. Vehicle: 10% glycerol-PBS. Differences between 5G2 and control IgG were analysed using ANOVA. (**c**) Immunohistochemistry of sectioned CTOSs with 5G2 mAb (green) and ZO-1 (red). CTOSs were cultured in floating or gel embedded conditions. Gel embedded CTOSs were cultured for 3 days in type I collagen gel. Scale bar, 50 μm. (**d**,**e)** Effect of pre-incubation with 5G2 mAb on CTOS growth under Matrigel embedded **(d**) or floating (**e**) culture conditions. Growth of CTOSs was evaluated by the day 7 to day 0 size ratio and the data normalized to a mean of 0 μg/ml for each experiment. Values are relative to the negative control (non-treated). Statistical analysis was performed by the Kruskal-Wallis test. Horizontal bar: median; boxes: 25^th^ and 75^th^ percentiles; bars: 10^th^ and 90^th^ percentiles. Data are from more than three independent experiments. V, vehicle, 10% glycerol-PBS.

To generate antibodies that recognize molecules on the CTOS surface, we intraperitoneally injected colorectal CTOSs, C45, into a mouse without adjuvant, expecting to preserve the 3D structure. Generation of antibodies against the CTOS surface was monitored in mouse serum by whole mount immunocytochemistry (ICC) without permeabilization after several boost injections (Fig. 1a). Spleen cells were then fused to myeloma cells to obtain hybridomas. The culture media from each hybridoma was screened by whole mount ICC of CTOS without permeabilization, and 90 clones were selected as generating antibodies against molecules on the CTOS surface.

These 90 hybridoma clones were evaluated for their effect on the adhesion of CTOSs to the extracellular matrix (ECM). Only clone 5G2 significantly inhibited CTOS adhesion to type I collagen-coated dishes in a dose-dependent manner (Fig. 1a,b). Inhibition of adhesion was also detected in Matrigel or type IV collagen-coated dishes (Fig. S1). Limiting dilution was performed twice to clone the 5G2 monoclonal antibody (mAb) and the isotype of purified antibody found to be IgG3κ. ICC with sectioned CTOSs revealed that the 5G2 mAb signal co-localized with apical membrane marker ZO-1 on the surface of CTOSs under floating conditions and on the surface inside the lumen in gel-embedded culture (Fig. 1c). 5G2 signals were also detected on the lateral membrane or in the cytosol, but the signal intensity was lower than at the apical membrane. Growth was inhibited by pre-incubation with a relatively high dose (100 μg/ml) of 5G2 antibodies under Matrigel embedded conditions (Fig. 1d), but not under floating conditions (Fig. 1e). These results indicate that 5G2 mAb mainly recognized molecules on the apical membrane of CTOSs and inhibited the adhesion of CTOSs to the ECM.

### 5G2 mAb inhibited adhesion and migration of colorectal cancer cells

**Figure 2:**
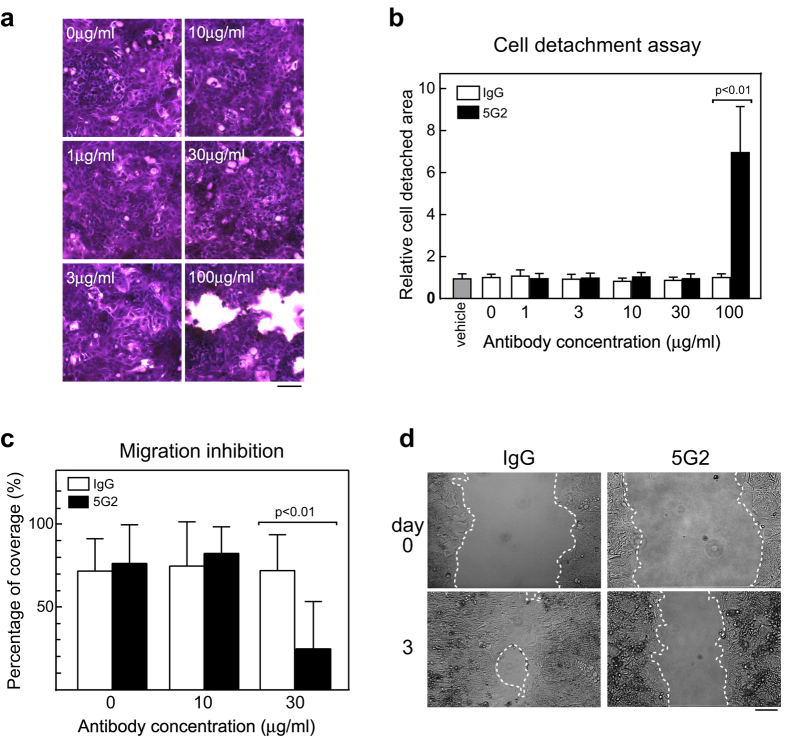
5G2 mAb promoted C45-2D cell detachment and inhibited cell migration on collagen-coated dishes. (**a**) Cresyl violet staining of C45-2D cells. Confluent C45-2D cells were incubated with 5G2 mAb for 3 days. Scale bar, 100 μm. (**b)** Quantification of cell detachment areas when treated with the indicated antibodies. Data indicate mean ± SD from three independent experiments. Differences were analysed by the Kruskal-Wallis test. (**c)** Quantification of cell migration. C45-2D cell migration was assessed by the percentage of closure after 3 days in culture after removal of the culture insert and antibody addition. Data indicate mean ± SD from four independent experiments. Differences were analysed by the Kruskal-Wallis test. (**d)** Representative images of the cell migration assay in (**c**). The antibody doses were 30 μg/ml. The white dotted lines indicate the edge of the space. Scale bar, 100 μm.

As 5G2 mAb inhibited the adhesion of CTOSs to the ECM, we further analysed the effect of 5G2 on cell adhesion and migration in detail. The C45 CTOSs were cultured on type I collagen-coated dishes (C45-2D cells). When 5G2 mAb was added to confluent C45-2D cells, cell detachment increased 6.9-fold at 100 μg/ml (Fig. 2a,b). The cell detachment was not likely due to direct cell toxicity, as TUNEL-positive cells were restricted at the edge of the remaining cells, probably because these cells peeled off when adjacent cell clusters detached (Fig. S2). In addition, detachment occurred as cell clusters rather than single cells, and the detached cell clusters eventually formed spheroids, indicating that 5G2 mAb inhibited adhesion to the ECM but not cell-cell contact.

The addition of 5G2 mAb also inhibited the migration of C45-2D cells (Fig. 2c) with a 65.5% decrease at 30 μg/ml (Fig. 2d). The promotion of cell detachment by the addition of 5G2 mAb indicated that blocking antigen molecules with 5G2 mAb disrupted already established adhesion. The inhibition of migration also indicated that 5G2 mAb inhibited novel adhesion formations consistent with adhesion inhibition.

### Patient tumours and CTOSs but not cell lines expressed 5G2 antigen

**Figure 3:**
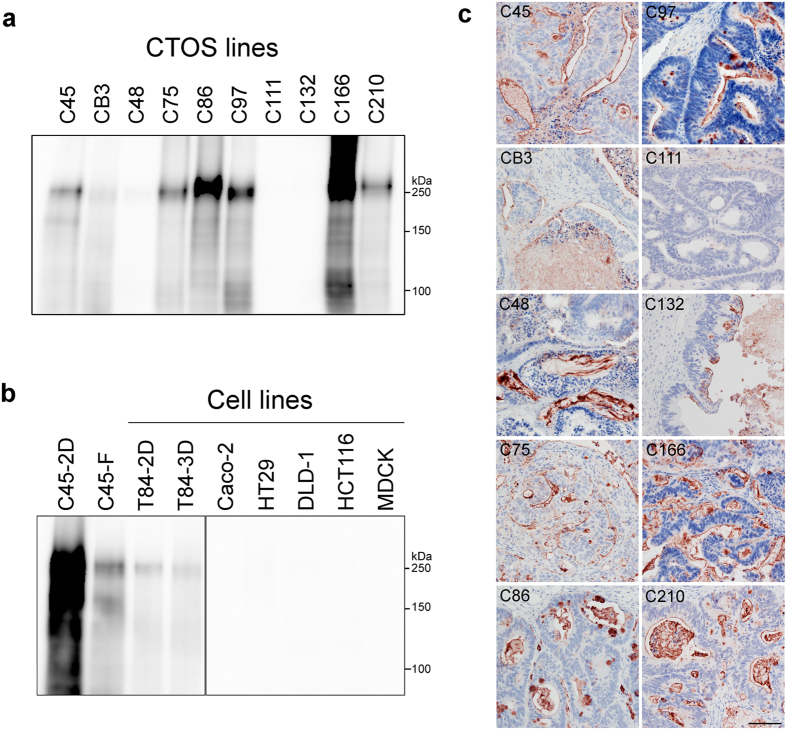
Expression of 5G2 mAb antigen in colorectal cancer-derived CTOSs and cell lines. **(a**,**b)** Western blot of lysate from colorectal cancer-derived CTOSs (**a**) and cell lines (**b**) using 5G2 mAb. (**c)** Immunohistochemical analysis of CTOS-derived xenotumour with 5G2 mAb. Scale bar, 100 μm.

To assess whether the 5G2 antigen is expressed in other colorectal CTOSs and cell lines, the expression of 5G2 mAb antigen was analysed using 10 colorectal CTOS lines and 7 conventional cell lines. Many of the CTOS lines expressed 5G2 antigen at various levels (Fig. 3a), but most of the cell lines did not express 5G2 antigen at all (Fig. 3b). Only T84 cells, a relatively differentiated cell line, had weak signal; polarity-maintaining MDCK cells did not express 5G2 antigen. Different expression levels in CTOSs and cell lines were not likely due to 2D or 3D culture conditions, because 5G2 expression levels in T84 cells did not increase under 3D conditions, and the expression levels in C45 CTOSs rather increased under 2D than 3D conditions (Fig. 3b).

Immunohistochemical analysis of xenotumours derived from colorectal CTOS lines also showed expression of 5G2 antigen. The 5G2 signal localized on the apical surface of the lumen at various levels (Fig. 3c). These results were consistent with the apical localization of the 5G2 signal in CTOSs cultured *in vitro* (Fig. 1c). The signal was also observed inside the lumen, suggesting that antigen molecules on the apical membrane may be secreted or shed into the lumen of the glandular structure. Thus, the 5G2 antigen was widely detected in CRC CTOSs and their xenotumours, but hardly in many conventional cell lines.

### 5G2 mAb recognized sugar chain epitopes

**Figure 4:**
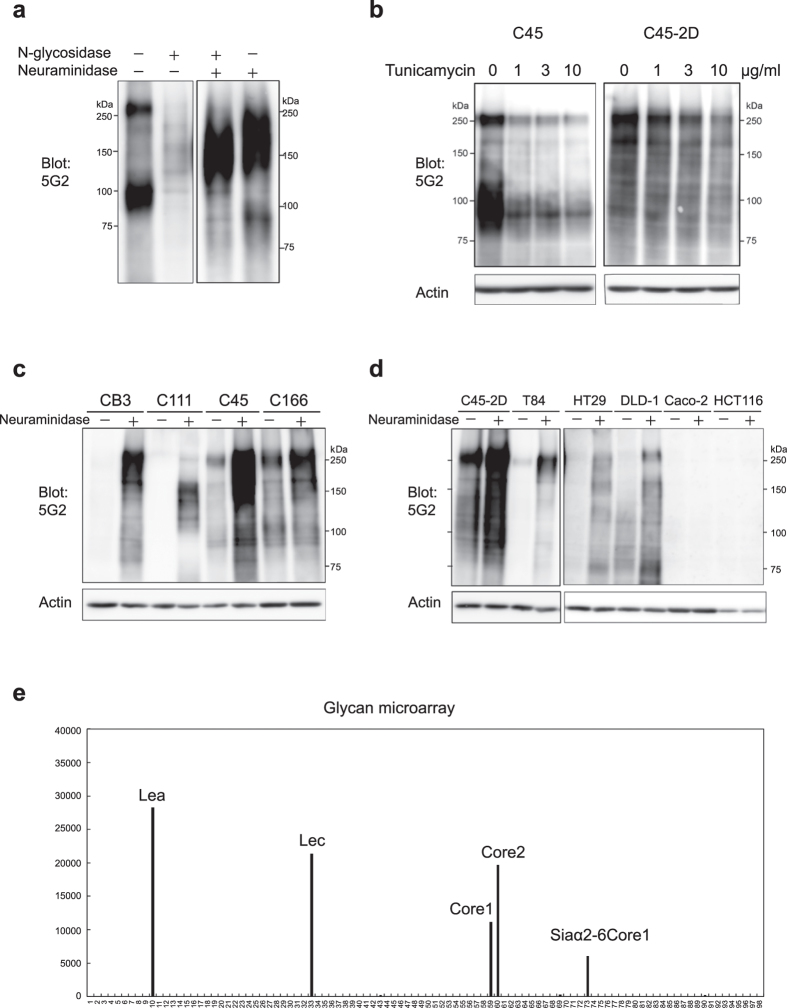
5G2 mAb recognized asialo-type N-glycan structure. (**a)** Western blot of glycosidase digested membrane fractions of C45 CTOSs using 5G2 mAb. **(b)** Western blot of lysate from tunicamycin-treated cells using 5G2 mAb. C45 CTOSs and C45-2D cells were cultured with the indicated doses of tunicamycin for 2 days. (**c**,**d)** Western blot of sialic acid-digested cell lysate using 5G2 mAb. Protein lysates from CTOSs (**c**) and cell lines (**d**) were digested by neuraminidase. Actin is shown as a loading control. (**e)** Glycan microarray for determination of the glycan structure recognized by 5G2 mAb. The names of glycan structures with high affinity are indicated.

Western blotting with 5G2 mAb revealed broad and smeared bands, suggesting that the 5G2 antigen recognizes glycosylated molecules. To identify antigen proteins, the molecular weights of the antigen molecules without glycosylation were examined. Membrane-rich fractions of C45 were digested with N-glycosidase and/or neuraminidase. Surprisingly, the 5G2 signals disappeared after the digestion of N-glycan by N-glycosidase, and additional digestion of sialic acid by neuraminidase restored the signal (Fig. 4a). Single digestion by neuraminidase also increased 5G2 reactivity compared to the un-digested sample. No sharp bands caused by un-glycosylated protein appeared after the digestion of glycan. These results suggest that the 5G2 mAb recognizes the N-glycan moiety and modification with sialic acid inhibits 5G2 recognition. The intense signal with double digestion of N-glycosidase and neuraminidase indicated that the masked antigen by sialic acids was abundant in C45 CTOSs. The intensity of the signal was compatible with or without N-glycosidase treatment, indicating that the masked antigen was mainly on O-glycan. The size of the bands treated with double digestion was smaller than that with neuraminidase single digestion, probably because the masked antigen on N-glycan was lost by N-glycosidase treatment.

To confirm that 5G2 recognized N-glycan, C45 CTOSs and C45-2D cells were treated with tunicamycin, an inhibitor of N-glycan synthesis. The reactivity of 5G2 mAb was decreased in both (Fig. 4b). The decrease of 5G2 signal was observed in relatively low dose (1μg/ml), although it is still possible that the decrease was also due to general suppression of protein synthesis by tunicamycin.

Various CTOSs and cell lines were analysed for their reactivity to 5G2 mAb with neuraminidase digestion. All of the CTOS lines had more intense 5G2 signals than the undigested samples, even if it was not detected without neuraminidase digestion, as in CB3 and C111 (Fig. 4c). The 5G2 signal appeared after neuraminidase digestion in the HT29 and DLD-1 cell lines, but not the Caco-2 and HCT116 cell lines (Fig. 4d). These results suggest that 5G2 antigen glycan was commonly further modified with sialic acid, and the modification inhibited recognition by 5G2.

The glycan binding property of 5G2 mAb was investigated using a glycan microarray. The 5G2 mAb recognized the Le^a^ (Galβ1-3(Fucα1-2)GlcNAc), Le^c^ (Galβ1-3GlcNAc), core1 (Galβ1-3GalNAc), core2 (Galβ1-3(GlcNAcβ1-6)GalNAc), and Siaα2-6core1 (Siaα2-6(Galβ1-3)GalNAc) structures (Fig. 4e, Supplementary Table S1). Core1, core2, and Siaα2-6Core1 were O-glycan-specific structures. On the other hand, both N-glycan and O-glycan could be modified by Le^a^ and Le^c^. Furthermore, sialy-Le^a^ and sialy-Le^c^ were not detected by 5G2 mAb, confirming that the 5G2 mAb specifically recognized non-sialylated, asialo-type Le^a^ and Le^c^. These results suggest that the main antigens of 5G2 mAb were the Le^a^ and Le^c^ structures on N-glycan in CTOSs, and 5G2 mAb recognizes Galβ1-3GlcNAc/GalNAc as a minimum recognition epitope. Le^a^ and Le^c^ could exist on various glycan structures. Consistently, broad smear was observed in long exposure of Western blotting of C45 lysate with 5G2 mAb. Meanwhile, the 5G2 mAb revealed two intense bands in short exposure (Fig. S3). Therefore, C45 could express two major carrier glycoproteins modified with glycans containing the Galβ1-3GlcNAc/GalNAc epitope, such as Le^a^ and Le^c^, without further modification with sialic acid.

### The major antigens of 5G2 mAb were CEACAM5 and CEACAM6

The membrane-concentrated fraction of C45 was immunoprecipitated with the 5G2 mAb and then subjected to mass spectrometry analysis after SDS-PAGE. Lysate from HCT116 cells was used as a negative control. Several candidate proteins were selected based on peptides in the immunoprecipitates of C45 with 5G2 mAb but not negative control IgG. Peptides in HCT116 cells were excluded. Furthermore, candidate proteins were narrowed down based on higher expression levels in C45 than HT29 in microarray analysis (Supplementary Table S2).

**Figure 5:**
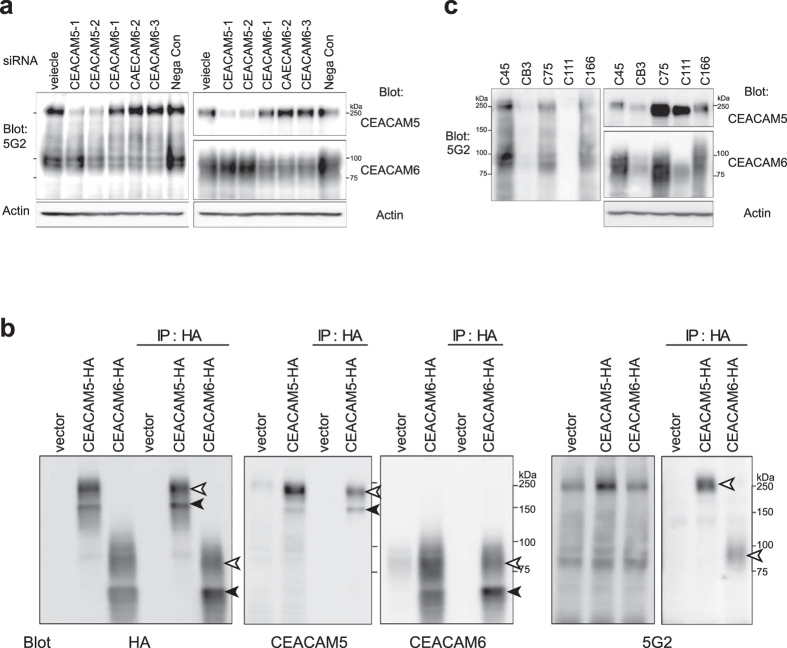
CEACAM5 and CEACAM6 were major carriers of 5G2 antigens. **(a)** Western blot of lysate from siRNA-transduced C45 CTOSs using the indicated antibodies. Actin is shown as a loading control. **(b)** Western blot of lysate from T84 cells in which HA-tagged CEACAM5 and CEACAM6 were forcibly expressed. Cell lysates were immunoprecipitated by HA antibody and detected by HA, CECAM5, CEACAM6, or 5G2 antibodies. The two major bands are indicated by arrowheads. **(c)** Western blot of lysate from colorectal cancer CTOS lines with CEACAM5, CEACAM6, and 5G2 antibodies. Actin is shown as a loading control.

For these candidates, 5G2 reactivity was examined using each siRNA in C45 CTOSs. 5G2 reactivity dramatically decreased when the expression of two of the candidates, CEACAM5 (known as CEA) and CEACAM6, was substantially repressed by siRNA (Fig. 5a). Further confirmation was obtained with over-expression of HA-tagged CEACAM5 and CEACAM6 in T84 cells, which had endogenously low but detectable 5G2 reactivity. HA-tagged CEACAM5 and CEACAM6 were immunoprecipitated and detected with 5G2 mAb, indicating that they contain the glycan structure recognized by 5G2. Antibodies against HA, CEACAM5, or CEACAM6 detected two major bands, whereas 5G2 mAb detected only upper bands, indicating that recognition by 5G2 mAb required full glycan modifications (Fig. 5b). These results suggest that the 5G2 mAb mainly recognized the N-glycans on CEACAM5 and CEACAM6. However, the expression levels of CEACAM5 and CEACAM6 detected by each specific antibody did not correspond to those detected by 5G2 in CTOS lines (Fig. 5c), suggesting that only a part of CEACAM5 and CEACAM6 have the N-glycan modification recognized by 5G2 mAb. The expression levels of CEACAM5 and CEACAM6 were lower in cell lines than in CTOSs, which is consistent with their lower reactivity to 5G2 mAb (Fig. S4). Furthermore, hierarchical clustering analysis using microarray data revealed that expression levels of glycosylation-related enzymes in CTOSs were closely related to each other as well as their tumours of origin, but not cell lines (Fig. S5). Thus, not only CEACAM expression, but also the expression profile of glycosylation-related enzymes differed between CTOSs and cell lines.

**Figure 6:**
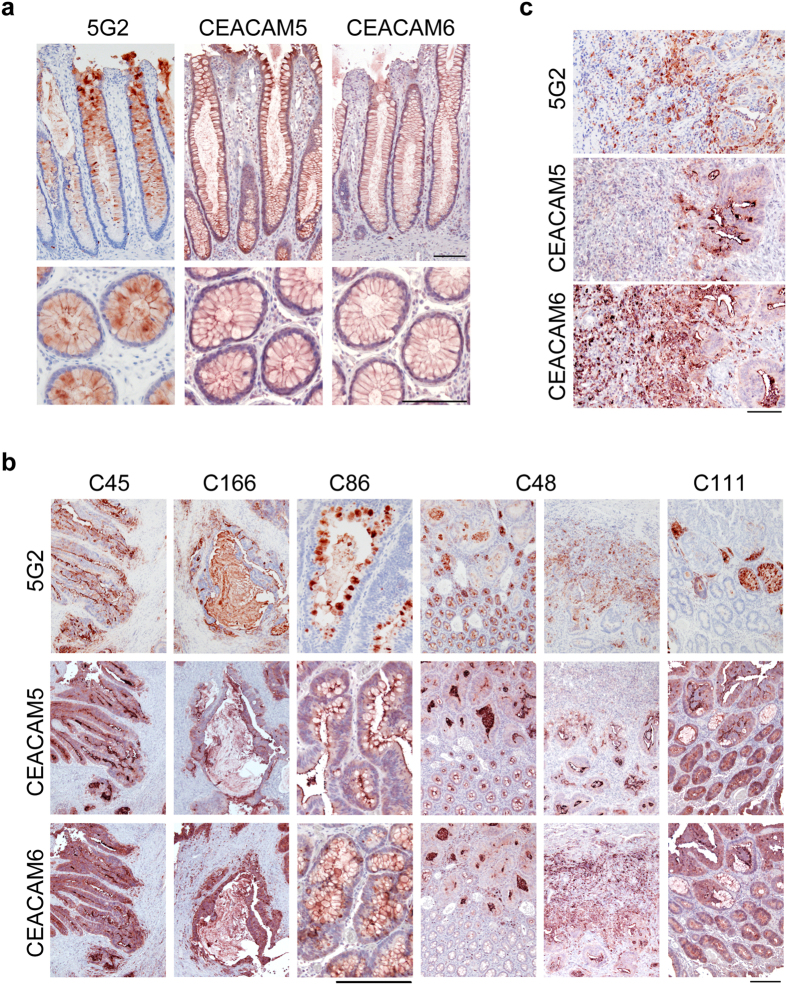
Localization of 5G2 antigen is consistent with CEACAM5 and CEACAM6 in patient tissues. (**a**) Immunohistochemical analysis of human normal colon and (**b**,**c**) tumour sections from patient tumours stained with CEACAM5, CEACAM6, and 5G2 antibodies as indicated. **(c)** High magnifications of the invasive front region of C48. Scale bar, 100 μm (**a,c**), 200 μm (**b**).

Immunohistochemical analysis of patient samples revealed that the 5G2 mAb signal was mostly matched with CEACAM5, CEACAM6, or both (Fig. 6). Similar results were observed in CTOS lines (Fig. S6). The 5G2 signal predominantly localized inside the cells rather than on the apical membrane in the normal colon (Fig. 6a). The 5G2 signal was also more predominant on the apical membrane and inside the lumen in patient tumours similar to CTOSs-derived xenotumours (Fig. 3c) compared to the CEACAM5 and CEACAM6 signal (Fig. 6b). The 5G2 signal preferentially co-localized with CEACAM6 at the invasive front of the C48 tumour (Fig. 6c). These results suggest that 5G2 mAb recognized the N-glycan on CEACAM5 and CEACAM6, which had a more secretory or invasion-related nature in tumours.

### 5G2 mAb inhibited integrin-related adhesion to the ECM

**Figure 7:**
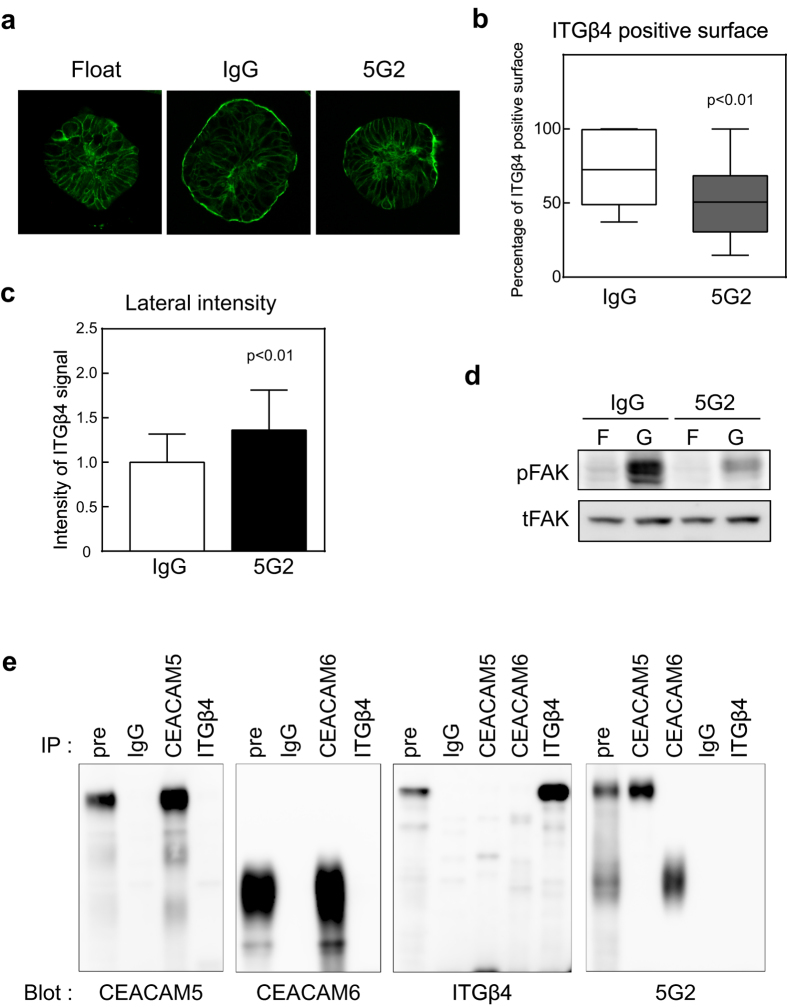
Pre-incubation with 5G2 mAb inhibited translocation of integrin β4 in CTOSs. **(a)** Immunocytochemical analysis of CTOSs using integrin β4 antibody. C45 CTOSs under floating conditions (i.e., suspension culture) were pre-incubated with the indicated antibody and cultured for 20 h in Matrigel. **(b)** CTOS surface coverage with the integrin β4 signal. C45 CTOSs were treated the same as in (**a**). Differences were analysed by the Mann Whitney U-test. IgG, n = 111; 5G2, n = 139. Horizontal bar, median; boxes, 25^th^ and 75^th^ percentiles; bars, 10^th^ and 90^th^ percentiles. **(c)** Integrin β4 signal intensity on lateral membranes in CTOSs. C45 CTOSs were treated the same as in (**a**). Data indicate mean ± SD. Differences were analysed by the Mann Whitney U-test. IgG, n = 1512; 5G2, n = 2066. **(d)** Western blot using phosphor-FAK (pFAK) or total FAK (tFAK) antibody. C45 CTOSs in floating conditions (F) were pre-incubated with the indicated antibody and cultured for 6 h in Matrigel (G). (**e**) Immunoprecipitation analysis of floating cultured C45 CTOSs. Antibodies used for immunoprecipitation (IP) and detection (Blot) are shown.

Next, we investigated the mechanism underlying 5G2 mAb inhibition of spheroid adhesion and the detachment of 2D cultured C45 CTOSs from collagen-coated dishes. Integrins are major ECM receptors. Microarray analysis revealed that, in C45, the expression levels of integrin β4 and α6 were higher than other integrin family members. Therefore, we focused on the relationship between 5G2 mAb and integrin β4. ICC revealed that integrin β4 localized mainly at the lateral membrane under floating conditions (Fig. 7a). When CTOSs were embedded in gel, integrin β4 translocated to the outer surface, which is the ECM interface, but treatment with 5G2 suppressed the translocation (Fig. 7a). Quantitative analysis of integrin β4 localization revealed that the average percent coverage of integrin β4 on the outer CTOS surface 20 h after Matrigel embedding significantly decreased from 70.6% (control IgG pre-incubation) to 50.8% with 5G2 pre-incubation (Fig. 7b). Furthermore, the intensity of integrin β4 signals on the lateral membrane was significantly greater in 5G2 pre-incubated CTOSs than in IgG pre-incubated CTOSs (Fig. 7c). These results suggest that 5G2 mAb impairs the translocation of integrin β4 from the lateral membrane to the ECM contact interface. Integrin binding with the ECM induces the phosphorylation of focal adhesion kinase (FAK)^24^. We found that FAK phosphorylation increased 6 h after Matrigel embedding, and 5G2 mAb pre-incubation suppressed FAK phosphorylation (Fig. 7d).

To confirm whether the inhibition of the integrin signal is due to direct interaction between CEACAMs and integrin β4, immunoprecipitation was carried out (Fig. 7e). Integrin β4 did not co-immunoprecipitate with CEACAM5 or CEACAM6, and neither CEACAM5 nor CEACAM6 co-immunoprecipitated with integrin β4, suggesting that CEACAM5 and CEACAM6 do not directly associate with integrin β4. In addition, 5G2 mAb did not recognize immunoprecipitated samples with integrin β4 antibody, indicating that integrin β4 does not have 5G2 antigen glycans.

These results suggest that pre-incubation with 5G2 mAb inhibited integrin signalling, probably through an attenuation of the translocation of integrin β4 from the lateral membrane to the ECM interface without direct interaction between CEACAM5 or CEACAM6 and integrin β4.

## Discussion

The CTOS is a culture system that preserves *in vivo* tumour characteristics. In this study, we generated monoclonal antibodies against the surface of CTOSs and identified a monoclonal antibody, clone 5G2, that inhibits CTOS adhesion to the ECM. We demonstrated that the antibody recognizes N-glycan structures, including Le^a^ and Le^c^. Major carrier proteins with these glycan structures are CEACAM5 and CEACAM6, which are over-expressed in CRC.

Most of the proteins have post-translational glycan modifications. Glycosylation is known to play roles in the regulation of cell motility, cell adhesion, and receptor activation of malignant cells^25^. Aberrant glycosylation is often observed on the surface of tumour cells^26^,^27^,^28^, and many tumour markers in clinical use are antibodies against glycans^27^. Recent glycomics studies have elucidated that cancer-specific structures and abnormal glycan distribution can be novel biomarkers of cancer^26^,^29^. However, it is difficult to reproduce glycan structures *in vitro* in conventional cell lines because glycan structures are prone to being affected by many factors, such as the expression levels of substrate proteins and glycosyltransferases and function of the Golgi apparatus^25^,^30^.

We previously reported that CTOS maintain the morphology of their original tumours^10^. Here we demonstrated that CTOS also maintain the glycan structures of their original tumours. Antigens of 5G2 mAb were glycans highly expressed in CTOSs similar to patient tumours, but 2D-cultured cell lines expressed low levels of the antigens. Even 2D-cultured CTOS-derived cells, C45-2D cells, exhibited altered patterns on Western blots with 5G2 mAb. These results suggest that glycosylation patterns or protein expression can quickly change under 2D culture conditions. As studies of glycans in tumours mostly depend on limited patient samples, evaluating a small amount of glycan structure is difficult, but we demonstrated that CTOS can be a novel platform for studying tumour glycans.

Immunohistochemical analysis of patient samples revealed that, in normal regions, 5G2 signals are observed mainly inside cells, whereas CEACAM signals localize on the membrane. In contrast, in tumour regions, 5G2 signals decrease inside cells but increase on the apical membrane and accumulate in the lumen, whereas CEACAM signals occur on the membrane and in the cytosol. The discrepant localization of 5G2 and CEACAM signals may be due to the 5G2 antigen being an immature form of the glycosylation on CEACAMs, which may not be transported to the apical membrane and remain inside the cell compartments, such as Golgi, in normal cells. In cancer cells, the protein transport system may be disturbed so that CEACAMs with 5G2 antigen glycan can be localized at the membrane and secreted into the lumen. The different pattern of localization of 5G2 and CEACAM signals in normal and tumour tissue suggests that 5G2 mAb could be a more tumour-specific marker, though further analysis is required.

Sialylation is enhanced in cancer cells^26^. CA19-9, one of the CRC tumour markers, recognizes sialyl-Le^a ^26. A small amount of Le^a^ and Le^c^ was previously reported to exist on CEA^31^. In this study, the asialo-type 5G2 antigen glycans on CEACAMs were also minor structures, as neuraminidase digestion significantly increased 5G2 reactivity. Asialo-type 5G2 antigen was highly expressed in CRC, probably because the expression levels of CEA proteins are increased, as well as N-glycosylation on CEA^32^. In-depth N-glycan profiling indicates that α2,3-sialylation is down-regulated in CRC tumours relative to non-tumorigenic tissues^33^; therefore, tumour cells may have incomplete α2,3-sialylated glycans of CEA, such as asialo-type 5G2 mAb antigen. In addition, a functional monoclonal antibody recognizing such minor glycan structures was generated, suggesting that 5G2 antigen glycan locates to an easily accessible CTOS surface for immune cells and the antibody.

Detailed analysis of the sialylation effect by the modification of specific sites on the glycan structure is difficult because CEACAM5 has 28 N-glycosylation sites, and neuraminidase digestion or neuraminidase mutant cells also affect other protein glycans. The CEACAM family consists of 22 different genes, including 12 CEACAM subgroup genes and 10 pregnancy-specific glycoproteins (PSG). The amino acid sequences reveal homology in the immunoglobulin (Ig) variable-like domain (IgV-like) and Ig constant C2-like domain (IgC2-like) among their extracellular domains^34^,^35^,^36^, suggesting that other minor CEA proteins can also be modified with 5G2 antigen glycan. In addition, as the core glycan structure of 5G2 recognition was revealed to be Galβ1-3GlcNAc/GalNAc according to the glycan array analysis, we do not exclude the possibility that the antigens are not specific to CEACAM5 and 6. Thus, detailed characterization of the role of 5G2 antigen glycan requires further analysis.

CTOSs may be able to metastasize because they resemble CTC clusters, which are highly associated with metastatic potential^14^,^15^,^16^. Recently, we reported that colorectal CTOSs have an apical membrane on the outer surface of the spheroid when cultured under floating conditions, and the polarity promptly switches when they are embedded in the ECM, forming lumens surrounded by the apical membrane inside the spheroids (Okuyama, Am J Pathol, in press). In the region of microvessel invasion in patient tumours, some of the cell clusters are surrounded by an apical membrane, indicating that floating CTOS-like cell clusters can be an origin of metastasis. In this scenario, the first step at the metastasis site is contact between the apical membrane and the vessel surface, and then invasion of the ECM and polarity switching to form metastatic foci that have the same polarity status as the original tumours. The process of floating spheroids becoming monolayer cells mimics the metastatic process, including migration, invasiveness, chemoresistance, and expression of cancer stem cell markers^37^,^38^. As 5G2 mAb inhibited the adhesion of floating CTOSs to the ECM, the antibody may have the ability to inhibit metastasis.

We showed that pre-incubation of the spheroids with 5G2 impaired translocation of integrin β4 from the lateral membrane to the contact interface between the extracellular matrix when embedded in it. As the integrin is known to play critical role on cell adhesion to extracellular matrix, the impairment of the integrin β4 translocation is likely to be involved in the adhesion inhibition, although the detailed mechanism remains unclear. Overexpression of CEACAM5 and CEACAM6 reportedly increase the binding ability of integrin α5β1 to fibronectin^39^. Heterodimerization of CEACAMs by crosslinking with antibody enhances the adhesion of neutrophils to fibronectin, accompanied by increased localization of Src family kinase to the cytoskeleton and increases their kinase activity^40^, and antibody-mediated crosslinking of CEACAM6 induces resistance to anoikis through Src-mediated FAK phosphorylation in a caveolin-1-dependent manner^41^. Taken together, these reports suggest that clustering via the extracellular domains of GPI-anchor type CEACAM5 and CEACAM6 regulates adhesion to the ECM through integrin signalling. 5G2 mAb may inhibit the clustering by recognizing N-glycan on the extracellular domain of CEACAM5 and CEACAM6, inhibiting adhesion to the ECM and delaying integrin translocation to the ECM contact interface, resulting in reduced FAK phosphorylation.

Although we focused on the 5G2 clone in detail in this study, we also obtained many other antibodies that recognize the CTOS surface and may be antibody-mediated drugs targeting CTC clusters, as the existence of apical membrane inside the vessel is abnormal. As the ability of the antibodies to internalize is critical for antibody-mediated targeting^42^,^43^, further screening by internalization may be useful for finding antibody-mediated drug candidates. In the case of the antigen proteins being secreted or shed from the apical membrane, they can be tumour markers.

## Methods

### Generation of monoclonal antibodies

C45 CTOSs were washed and suspended in HBSS (GIBCO). A 10-week-old female Balb/c mouse (CLEA Japan, Inc., Tokyo, Japan) was immunized by intraperitoneally injecting 1–2.5 × 10^4^ CTOSs. A boost injection was performed eight times at 1-week intervals. Three days after the final boost injection, spleen cells were harvested and fused to mouse myeloma cells (P3-X63Ag8.653) using polyethylene glycol (Sigma, St. Louis, MO). The fused cells were cultured with CM-B medium (EIDIA, Tokyo, Japan) and hybridoma cells selected by HAT selection. Clone 5G2 was cloned by limiting dilution (twice) and expanded with SF-B medium (EIDIA). Antibody was purified using the MAbTrap kit (GE Healthcare, Buckinghamshire, UK). The immunoglobulin class was determined using the IsoStrip mouse monoclonal antibody isotyping kit (Roche Diagnostics GmbH, Mannheim, Germany).

### CTOS preparation and cell culture

The collection, handling, and use of human tumour tissue samples were performed in accordance with protocols approved by the institutional ethics committees at Osaka Medical Center for Cancer and Cardiovascular Diseases. Human tissue samples were obtained after informed consent. All animal work was performed according to the protocols approved by the institutional animal study committee of Osaka Medical Center for Cancer and Cardiovascular Diseases. CTOSs were prepared and cultured according to a previously described protocol^10^. Briefly, CTOS-derived xenograft tumours were dissected from immunodeficient mice. After mechanical dissociation, tumour fragments were digested with Liberase (Roche) for 2 h at 37 °C. Cell clusters ranging in size from 40 μm to 250 μm were collected by filtration with steel wire mesh and cell strainers (Falcon, Corning, NY). Collected cell clusters were cultured in StemPro hESC (GICO, Waltham, MA) and maintained under floating culture conditions. Cell lines were obtained from American Type Culture Collection (ATCC, Manassas, VA). T84 cells were cultured in DMEM/F12 (GIBCO), HT29 cells and HCT116 cells in MacCoy’s 5a medium (GIBCO), DLD-1 cells in RPMI1640 medium (GIBCO), and Caco-2 cells and MDCK cells in MEM (GIBCO) with 0.1 mM NEAA (GIBCO), each supplemented with 10% FBS. C45-2D cells were established from C45 CTOSs (colorectal cancer-derived). CTOSs were digested with trypsin, plated in type I collagen-coated dishes, and cultured in DMEM/F12 supplemented with 10% FBS.

### Adhesion assay

Approximately 100 CTOSs were incubated with hybridoma-cultured media or purified antibody at 37 °C for 3 h, and then plated in 96-well tissue culture plates (IWAKI Shizuoka, Japan) coated with type I collagen (Nitta Gelatin Inc., Osaka, Japan). The total number of and number of adhered COTSs were counted on day 0 and day 2, respectively.

### Detachment assay

C45-2D cells were cultured on collagen-coated 96-well culture plates until reaching confluence. Antibodies were added to the cells and cultured for 3 days. Cells were fixed with 4% PFA and stained with cresyl violet. Three independent field images were taken of one well and the cell-free area measured by ImageJ (National Institutes of Health, Bethesda, MD). The experiments were performed in triplicate and repeated three times.

### Migration assay

C45-2D cells were cultured on type I collagen-coated 8-well μ-slide with culture-insert (ibidi, Martinsried, Germany). After cells reached confluence, the insert was removed and the medium changed to 1% FBS containing DMEM/F12 with antibodies, and then cultured 3 more days. Photos were taken upon the removal of the insert and after 3 days in culture. Four photos were taken of one well. The cell-free area was measured by ImageJ. The experiments were repeated four times. The percentage of coverage equals the cell-free area on day 3/cell-free area on day 0 × 100.

### Growth assay

CTOSs 40–70 μm in size 5 to 8 days after preparation from xenografts were pre-incubated with antibodies at 37 °C overnight. The CTOSs were then transferred to a well on a 96-well plate and cultured for 1 week under floating or Matrigel (GIBCO) embedded conditions. Photos were taken with phase contrast microscopy and CTOS size measured by ImageJ. Growth was evaluated based on the day 7 to day 0 size ratio.

### Immunohistochemical analysis

CTOSs were fixed with formaldehyde and then embedded in paraffin. Paraffin sections (4 μm) were deparaffinized and antigen retrieval performed in 10 mM citrate buffer (pH 6.0) for 15 min at 121 °C. After blocking with 10% normal goat serum in PBS containing 0.1% Triton X-100 (PBS-TX), the sections were incubated with primary antibodies in 5% goat serum in PBS-TX at 4 °C overnight. For immunoperoxidase detection, the sections were incubated with biotin-conjugated secondary antibody (Vector Lab, Burlingame, CA), labelled using the VECTASTAIN Elite ABC kit (Vector), and visualized with NovaRED (Vector). For immunofluorescent detection, Alexa Fluoro 488- and 594-conjugated secondary antibody (Molecular Probes) was used. For integrin β4 staining, antigen retrieval was performed by treatment with 20 μg/ml proteinase K in 50 mM Tris-HCl (pH 8.0), 1 mM EDTA, and 0.1% Triton for 15 min at room temperature. After mounting with ProLong Gold antifade mountant with DAPI (Molecular Probes), images were obtained using a BX50 microscope (Olympus, Tokyo, Japan) with a DP73 CCD camera or confocal microscope (TCS SPE; Leica Microsystems, Wetzlar, Germany).

### Whole mount immunocytochemistry of CTOSs

C45 CTOSs were washed with 0.5% BSA in PBS and blocked with 1% BSA in PBS at room temperature for 15 min. The CTOSs were then incubated with hybridoma-cultured media at room temperature for 1 h. After washing three times, CTOSs were incubated with Alexa488-conjugated anti-mouse IgG (Molecular Probes, Waltham, MA) and Hoechst33342 (Molecular Probes) at room temperature for 1 h. Fluorescence images were captured using confocal microscopy.

### Antibodies

Antibodies against ZO-1 were purchased from Invitrogen; CEACAM5 from Abcam (Cambridge, UK); CEACAM6 from Santa Cruz Biotechnology (Dallas, TX); β-actin from Sigma; phosphor-FAK from Invitrogen; FAK from BD Bioscience (San Jose, CA); and integrin β4 from Novous Biological (Littleton, CO).

### Western blotting

CTOSs were lysed with RIPA buffer (10 mM Tris-HCl pH 7.4, 1 mM EDTA, 150 mM NaCl, 1% NP40, 0.5% sodium deoxycholate, 0.1% SDS) containing EDTA-free complete proteinase inhibitor (Roche) and PhosSTOP phosphatase inhibitor (Roche) at 4 °C. After sonication and centrifugation, the supernatant was collected. The protein concentration was measured using the BCA protein assay kit (Pierce, Waltham, MA). Basically, 10 μg of protein for detection of phosphorylation, 20 μg of protein was applied to one lane. After electrophoresis and blotting, the blots were blocked with 5% BSA in TBS-T (20 mM Tris-HCl pH 7.6, 150 mM NaCl, 0.1% Tween-20) at room temperature, and then incubated with primary antibody in TBS-T containing 1% BSA overnight at 4 °C. After washing with TBS-T, the blots were incubated with HRP-conjugated secondary antibody (Santa Cruz) in TBS-T containing 5% (w/v) skim milk for 1 h at room temperature. After washing with TBS-T, signal was detected using ECL Prime Western blotting detection reagent (GE Healthcare) with an LAS-4000mini (GE Healthcare).

### Immunoprecipitation

Cells or CTOSs were homogenized and lysed with TNE-T buffer (10 mM Tris-HCl pH 7.4, 1 mM EDTA, 150 mM NaCl, 1% Triton) containing EDTA-free complete proteinase inhibitor (Roche) at 4 °C. After centrifugation at 14,000 rpm for 15 min at 4 °C, the supernatant was collected. After pre-clearing with protein G sepharose (GE Healthcare) for 1 h at 4 °C, the lysate was incubated with antibodies at 4 °C overnight, and then the antibodies collected by incubation with protein G sepharose. After washing with TNE-T buffer, immunoprecipitated proteins were eluted by heating in 2x SDS-PAGE sample buffer.

### Glycan analysis

Glycosylation was digested according to the protocol provided by the manufacturer of glycosidase enzymes. Briefly, protein samples were incubated in glycoprotein denaturing buffer with protease inhibitor cocktail at 100 °C for 10 min before treatment with glycosidase. N-glycosidase F (NEB, Ipswich, MA) and neuraminidase (NEB) were added to the denatured proteins and incubated at 37 °C overnight. N-glycan synthesis was inhibited by tunicamycin (Sigma). Glycan array analysis was performed according as described previously^44^. Briefly, 5G2 mAb was pre-complexed with Cy3-labelled anti-mouse IgG antibody and then applied to the glycan array at 10μg/ml. Fluorescent images were acquired using an evanescent field-activated fluorescent scanner.

### Plasmid construction and gene transduction

CEACAM5 and CEACAM6 cDNAs prepared from C45 CTOSs were amplified by PCR and the PCR products cloned into the TOPO cloning system (Invitrogen). After sequencing, the cDNAs were transferred to pCAGGS and 3x HA tag sequences inserted downstream of the signal sequence. Plasmid constructs were transfected into cells using X-tremeGENE HP DNA transfection reagent (Roche). For RNAi experiments, C45 CTOSs were incubated with 5 mM EDTA/PBS for 30 min at room temperature, followed by transduction of 150 pmole of Silencer select siRNAs (ABI, Waltham, MA) into 5000 CTOSs (40–70 μm) using a NEPA21 electroporator (Nepa Gene, Chiba, Japan). Three days after transduction of siRNAs, CTOSs were harvested and subjected to Western blotting.

### Statistical analysis

Differences between two groups were analysed by a two-tailed Mann Whitney U test for non-parametric data, and more than three groups were analysed by the ANOVA test for parametric data or Kruskal-Wallis test for non-parametric data. Each difference was determined by Tukey’s multiple comparison test for parametric data or Dunn’s multiple comparison test for non-parametric data. Statistical analyses were performed using GraphPad Prism 6 (GraphPad Software, San Diego, CA).

## Additional Information

**How to cite this article**: Sato, Y. *et al.* Generation of a monoclonal antibody recognizing the CEACAM glycan structure and inhibiting adhesion using cancer tissue-originated spheroid as an antigen. *Sci. Rep.* **6**, 24823; doi: 10.1038/srep24823 (2016).

## Supplementary Material

Supplementary Information
