# Corrigendum: Generation of a monoclonal antibody recognizing the CEACAM glycan structure and inhibiting adhesion using cancer tissue-originated spheroid as an antigen

**DOI:** 10.1038/srep26575

**Published:** 2016-05-31

**Authors:** Yumi Sato, Hiroaki Tateno, Jun Adachi, Hiroaki Okuyama, Hiroko Endo, Takeshi Tomonaga, Masahiro Inoue

Scientific Reports 6: Article number: 2482310.1038/srep24823; published online: 04212016; updated: 05312016

This Article contains a typographical error in the Results section under subheading ‘5G2 mAb recognized sugar chain epitopes’,

“The 5G2 mAb recognized the Le^a^ (Galβ1-3(Fucα1-2)GlcNAc), Le^c^ (Galβ1-3GlcNAc), core1 (Galβ1-3GalNAc), core2 (Galβ1-3(GlcNAcβ1-6)GalNAc), and Siaα2-6core1 (Siaα2-6(Galβ1-3)GalNAc) structures (Fig. 4e, Supplementary Table S1).”

should read:

“The 5G2 mAb recognized the Le^a^ (Galβ1-3(Fucα1-4)GlcNAc), Le^c^ (Galβ1-3GlcNAc), core1 (Galβ1-3GalNAc), core2 (Galβ1-3(GlcNAcβ1-6)GalNAc), and Siaα2-6core1 (Siaα2-6(Galβ1-3)GalNAc) structures (Fig. 4e, Supplementary Table S1).”

