# Supplementary material for: Generation of a monoclonal antibody recognizing the CEACAM glycan structure and inhibiting adhesion using cancer tissue-originated spheroid as an antigen
Source: Sci Rep. 2016 Apr 21;6:24823. doi: 10.1038/srep24823 (PMC4838943; doi:10.1038/srep24823)
Supplement: Supplementary Information [file srep24823-s1.pdf]

Supplementary information

Title : Generation of a monoclonal antibody recognizing the CEACAM glycan structure and inhibiting adhesion using cancer tissue-originated spheroid as an antigen

Authors : Yumi Sato[1,2], Hiroaki Tateno[3], Jun Adachi[4], Hiroaki Okuyama[1], Hiroko Endo[1], Takeshi Tomonaga[4], Masahiro Inoue[1*]

Affilations:

[1]Department of Biochemistry Osaka Medical Center for Cancer and Cardiovascular Diseases, Osaka, Japan, [2]Department of Molecular Virology and Oncology, Cancer Research Institute, Kanazawa University, Kanazawa, Japan, [3]Biotechnology Research Institute for Drug Discovery (BRD), National Institute of Advanced Industrial Science and Technology (AIST), Ibaraki, Japan, [4]Laboratory of Proteome Research, National Institute of Biomedical Innovation, Health and Nutrition, Osaka, Japan

Preparation of membrane rich fraction for glycosidase digestion

CTOSs were washed with PBS and homogenized in 9 volumes (w/v%) of homogenizing buffer (0.25M sucrose, 5mM Tris-HCl, pH7.4 with cOmplete, EDTA free proteinase inhibitor cocktail (Roche)), and then centrifuged at 1000×g for 10min at 4˚C. The supernatants (sup.1) were centrifuged at 10,000×g for 10min at 4˚C, and the resultant supernatants were further centrifuged at 105,000×g for 60min at 4˚C. The pellets were re-suspended to suspension buffer (50mM Tris-HCl, pH 7.4 with cOmplete, EDTA free) to make membrane rich fraction and subjected to glycosidase digestion analysis.

IP-MAS analysis

Sup.1 described above were centrifuged at 105,000×g for 1h at 4˚C, and the pellets were re-suspended to suspension buffer with Triton (20mM Tris-HCl, pH 8.0 with cOmplete, EDTA free proteinase inhibitor, 1% Triton) and incubated at 4˚C. Solubilized proteins were collected as supernatant of centrifugation (14,000 rpm for 15min at 4˚C), and diluted to five times with 20mM Tris-HCl, pH8.4. Unbound fraction to anion exchange resin (Bio-Rad Lab. Inc., Hercules, CA) was collected, and pre-cleared by incubation with mouse IgG3 kappa and Protein G sepharose (GE Healthcare), then incubated with 5μg/ml of 5G2 mAb at 4˚C for overnight. Immunoprecipitate was collected by incubation with protein G sepharose, and subjected to SDS-PAGE after boiling in

SDS-PAGE sample buffer. The gel was stained with Simple Blue Safe Stain (Invitrogen), cut out by molecular weight upper than 75kDa, and applied to in-gel digestion[1] and LC-MS/MS analysis. Digested peptides were analyzed using an LTQ-Orbitrap XL mass spectrometer (Thermo fisher scientific)[2]. The nano LC gradient is delivered at 500 nL/min and consists of a linear gradient of Buffer B developed from 5 to 35% B in 45 min. A spray voltage of 2000 V is applied. MS data was searched against human uniprot database using Proteome Discoverer 1.3 and Mascot v2.4. The precursor mass tolerance is set to 7 ppm and a fragment ion mass tolerance is set to 0.6 Da for CID. The search parameters allow for one missed cleavage for trypsin, fixed modifications (carbamidomethylation at cysteine), and variable modifications (oxidation at methionine, acetylation at protein N-terminal). Peptides identified at a threshold with 1% FDR are accepted. Candidates of antigen proteins were selected, which detected 8 times more than HCT116 derived samples, and not detected in IgG control (Table S2).

Microarray analysis

Total RNA was extracted from CTOSs or tumours with TRIzol reagent (Life Technologies) according to the manufacturer's instructions. One microgram of total RNA was reverse-transcribed to obtain cDNA using Superscript III (Life Technologies). Microarray hybridizations were performed at Hokkaido System Science (Sapporo,

Japan) using SurePrint G3 Human GE 8x60K Ver.1 (Agilent Technologies, Santa Clara, CA). The microarray slides were scanned and the gene expression profiles were analyzed at Hokkaido System Science according to the manufacturer's protocol. Each dataset was standardized using the z-score transformation method. Microarray data can be viewed using the NCBI Gene Expression Omnibus (GEO). For hierarchical clustering analysis, microarray data for glycosylation-related enzyme genes were picked up and hierarchical clustering performed with Pearson correlation using average linkage clustering after $\log_2$-transformation by MeV.

1 Adachi, J., Kumar, C., Zhang, Y., Olsen, J.V., Mann, M. The human urinary proteome contains more than 1500 proteins, including a large proportion of membrane proteins. Genome Biol. 7, R80, doi:10.1186/gb-2006-7-9-r80 (2006)

2 Narumi, R. et al. A strategy for large-scale phosphoproteomics and SRM-based validation of human breast cancer tissue samples. J. Proteome Res. 11, 5311-5322, doi:10.1021/pr3005474 (2012)

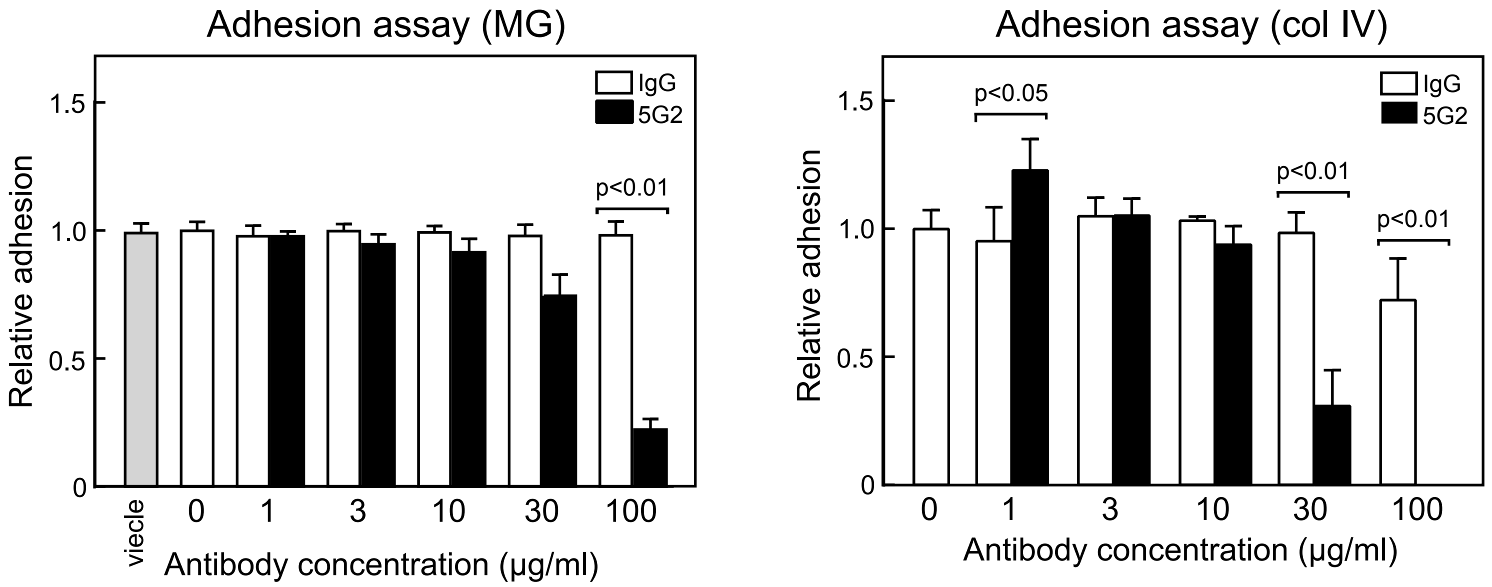

Supplemantary Figure S1

Quantification of adhesion to Matrigel (MG)- or type IV collagen (col IV)-coated plates.

Adhesion was evaluated as in Figure 1b. Data indicate mean ± SD from three independent experiments (MG) or a single experiment (col IV). Each condition was performed in triplicate and data are shown relative to the negative control (non-treated) in each experiment. Vehicle: 10% glycerol-PBS. Differences between 5G2 and control IgG were analysed by ANOVA.

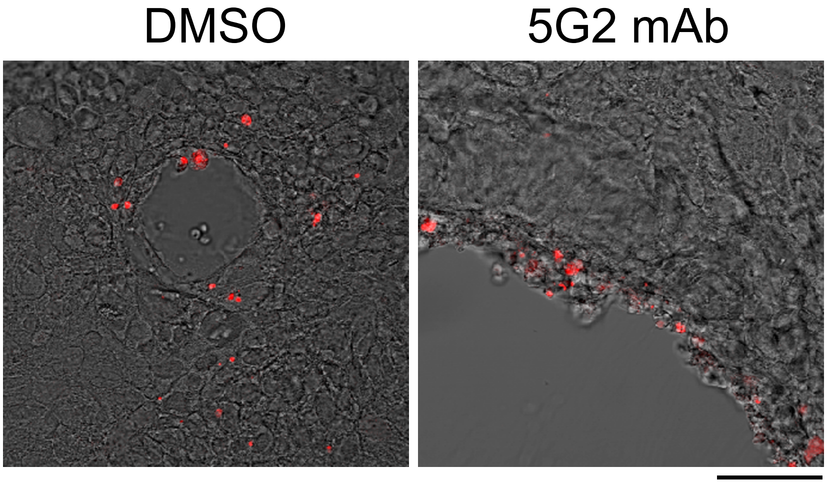

Supplementary Figure S2

Cell death induced by 5G2 mAb incubation were restricted at the edge of the cell detached area.
Death of C45-2D cells incubated with 5G2 mAb. 100 mg/ml 5G2 mAb or DMSO was added to confluent C45-2D cells and cultured for 2 days. After fixation with 1% PFA overnight, cell death was detected by TUNEL staining using the ApopTag Red In Situ Apoptosis detection kit (Millipore). Scale bar, 50 µm.

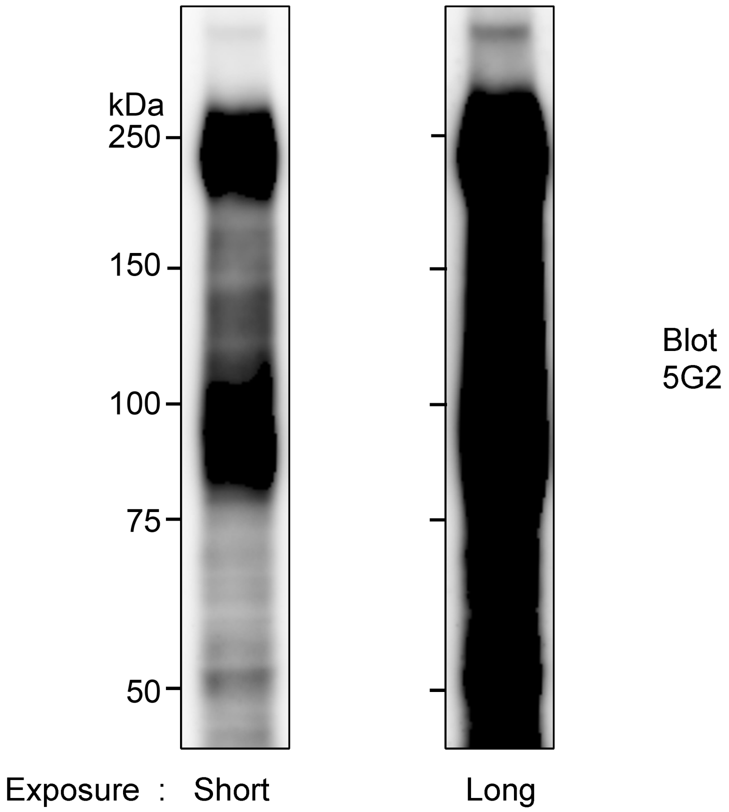

Supplementary Figure S3

5G2 mAb recognized various glycoproteins, including two major proteins.

Western blotting of lysate from floating cultured C45 with 5G2 mAb. Two major bands were detected in short (30sec) exposure and broad smear in long (120sec) exposure.

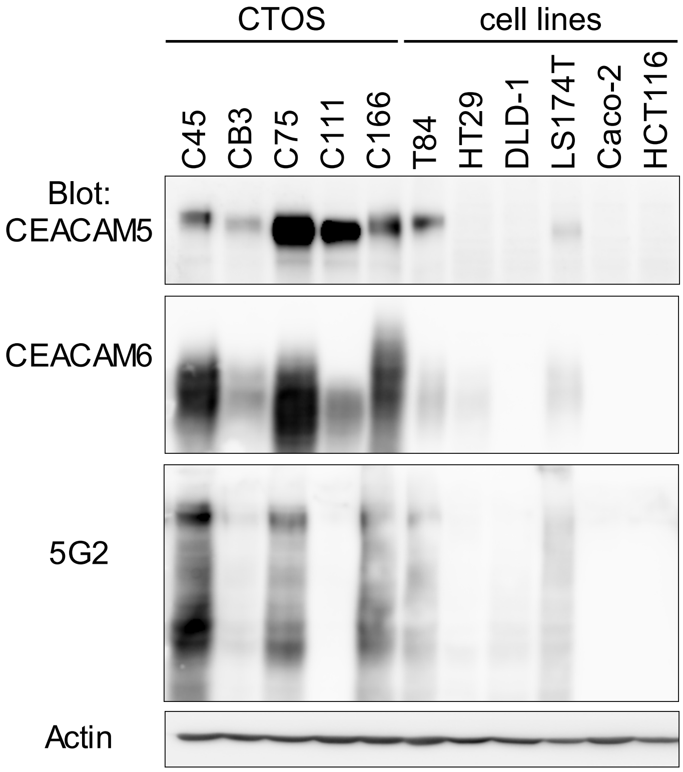

Supplementary Figure S4

Low levels of CEACAM5, CEACAM6, and 5G2 antigen expression in CRC-derived cell lines compared to CTOS lines. Western blotting of lysate from CRC-derived CTOSs and cell lines using CECAM5, CEACAM6, and 5G2 antibodies. Actin is shown as a loading control.

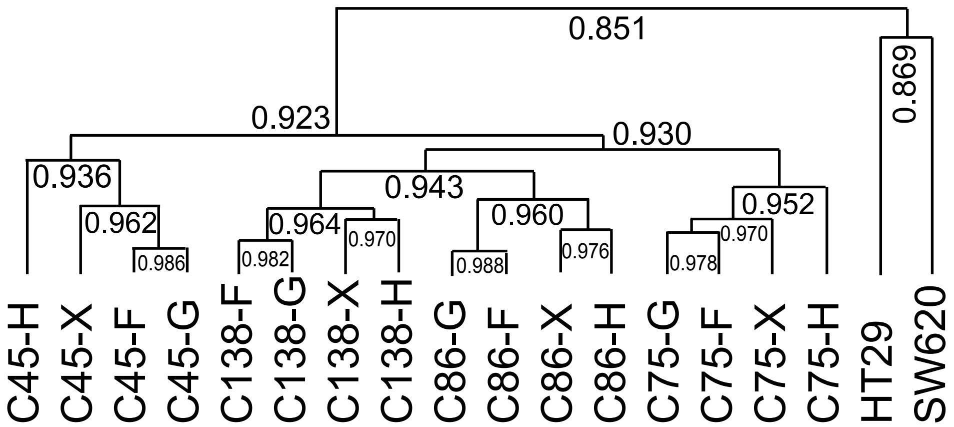

Supplementary Figure S5

Hierarchical clustering analysis of expression levels of glycosylation-related enzymes.

Numbers indicate distances. H, microdissected original tumors; X, CTOS-derived xenografts; F, CTOSs cultured under floating conditions; G, CTOSs cultured under gel-embedded conditions.

Supplementary Figure S6

Immunocytochemical analysis of CRC-derived CTOS lines with CEACAM5, CEACAM6, and 5G2 antibodies. CTOSs were cultured for 3 days in floating or cell matrix embedding conditions. Paraffin sections were stained with CEACAM5, CEACAM6, and 5G2 antibodies. Scale bar, 20 µm.

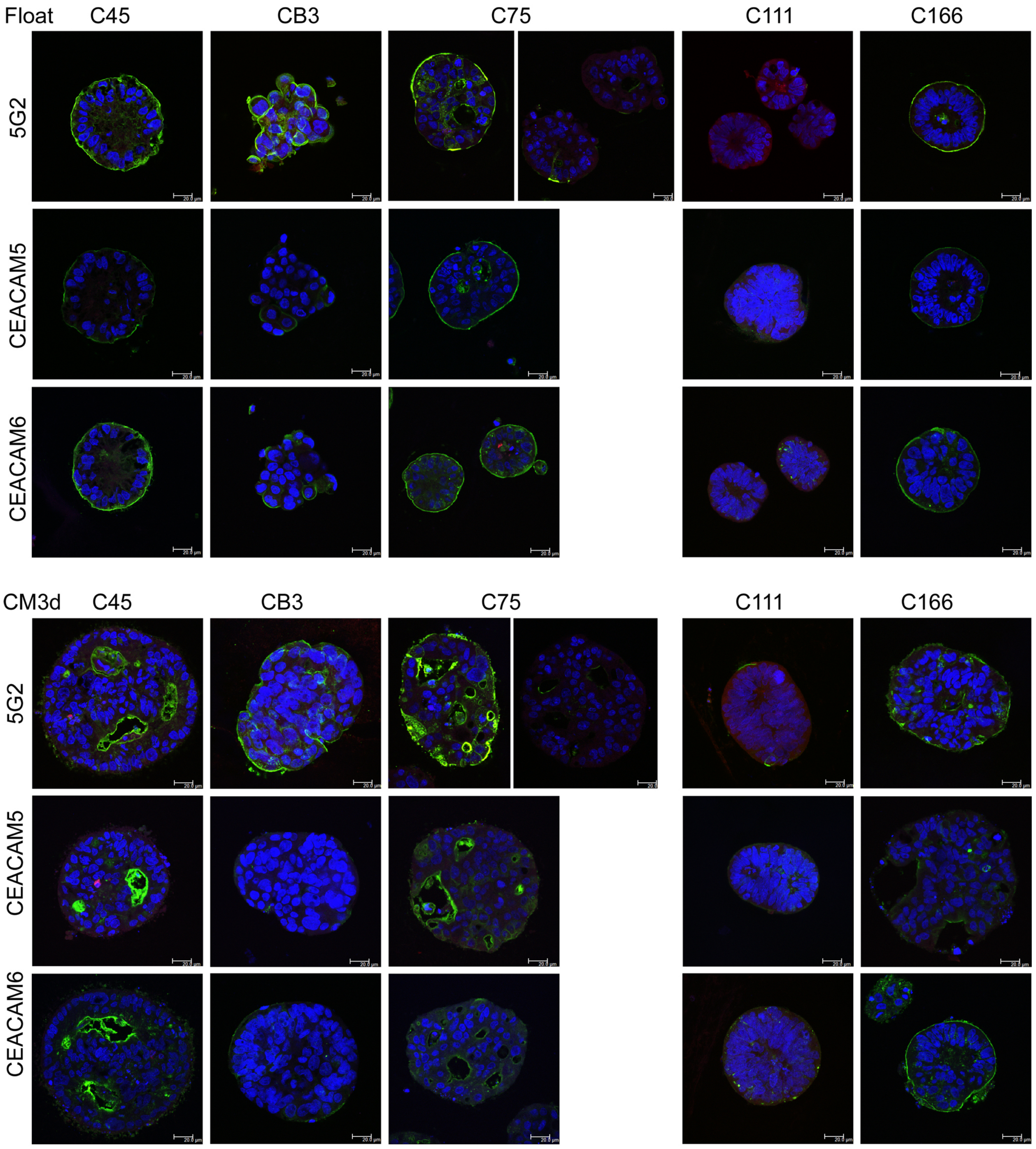

Supplementary Table.S1 Glycan microarray data

Number	Trivial name	Glycans	5G2 Precomplex 10ug/mL_14528-1
1	aFuc	Fuca1-PAA	0
2	Fuca2Gal	Fuca1-2Galb1-PAA	0
3	Fuca3GlcNAc	Fuca1-3GlcNAcb1-PAA	0
4	Fuca4GlcNAc	Fuca1-4GlcNAcb1-PAA	0
5	H type1	Fuca1-2Galb1-3GlcNAcb1-PAA	0
6	H type2	Fuca1-2Galb1-4GlcNAcb1-PAA	0
7	H type3	Fuca1-2Galb1-3GalNAca1-PAA	0
8	A	GalNAca1-3(Fuca1-2)Galb1-4GlcNAcb1-PAA	0
9	B	Gala1-3(Fuca1-2)Galb1-4GlcNAcb1-PAA	100
10	Le^a	Galb1-3(Fuca1-4)GlcNAcb1-PAA	28300
11	[3S]Le^a	$(3OSO_3)$Galb1-3(Fuca1-4)GlcNAcb1-PAA	0
12	Le^b	Fuca1-2Galb1-3(Fuca1-4)GlcNAcb1-PAA	0
13	Le^x	Galb1-4(Fuca1-3)GlcNAcb1-PAA	0
14	Le^y	Fuca1-2Galb1-4(Fuca1-3)GlcNAcb1-PAA	0
15	aNeu5Ac	Neu5Aca2-PAA	0
16	aNeu5Gc	Neu5Gca2-PAA	0
17	Sia2	Neu5Aca2-8Neu5Aca2-PAA	0
18	Sia3	Neu5Aca2-8Neu5Aca2-8Neu5Aca2-PAA	0
19	3'SiaLe^c	Neu5Aca2-3Galb1-3GlcNAcb1-PAA	0
20	3'SL	Neu5Aca2-3Galb1-4Glcb1-PAA	100
21	3'SLN	Neu5Aca2-3Galb1-4GlcNAcb1-PAA	100
22	sLe^a	Neu5Aca2-3Galb1-3(Fuca1-4)GlcNAcb1-PAA	0
23	sLe^x	Neu5Aca2-3Galb1-4(Fuca1-3)GlcNAcb1-PAA	0
24	6'SL	Neu5Aca2-6Galb1-4Glcb1-PAA	0
25	FET	Fetuin (Complex-type N-glycans and O-glycans)	0
26	AGP	a1-acid glycoprotein (Complex-type N-glycans)	0
27	TF	Transferrin (Complex-type N-glycans)	0
28	TG	Porcine thyroglobulin (Complex and high-mannnose-type N-glycans)	0
29	bGal	Galb1-PAA	0
30	[3S]bGal	$(3OSO_3)$Galb1-PAA	0
31	A-di	GalNAca1-3Galb1-PAA	0
32	Lac	Galβ1-4Glcb1-PAA	0
33	Le^c	Galb1-3GlcNAcb1-PAA	21400
34	[3'S]Le^c	$(3OSO_3)$Galb1-3GlcNAcb1-PAA	0
35	LN	Galb1-4GlcNAcb1-PAA	0
36	[3'S]LN	$(3OSO_3)$Galb1-4GlcNAcb1-PAA	0
37	[6S]LN	Galb1-4$(6OSO_3)$GlcNAcb1-PAA	0
38	[6'S]LN	$(6OSO_3)$Galb1-4GlcNAcb1-PAA	0
39	bGalNAc	GalNAcb1-PAA	0
40	di-GalNAcβ	GalNAcb1-3GalNAcb1-PAA	0
41	LDN	GalNAcb1-4GlcNAcb1-PAA	0
42	GA2	GalNAcb1-4Galb1-4Glcb1-PAA	0
43	Asialo-FET	Asialo fetuin (Desialylated complex-type N- and O-glycans)	200
44	Asialo-AGP	Asialo a1-acid glycoprotein (Desialylated complex-type N-glycans)	0
45	Asialo-TF	Asialo transferrin (Desialylated complex-type N-glycans)	0
46	Asialo-TG	Asialo porcine thyroglobulin (Desialylated complex-type N-glycans, high-mannose-type N-glycans)	0
47	βGlcNAc	GlcNAcb1-PAA	0
48	[6S]bGlcNAc	$(6OSO_3)$GlcNAcb1-PAA	0
49	Agalacto-Fet	Agalacto fetuin (Agalactosylated complex-type N- and O-glycans)	0
50	Agalacto-AGP	Agalacto a1-acid glycoprotein (Agalactosylated complex-type N-glycans)	0
51	Agalacto-TF	Agalacto transferrin (Agalactosylated complex-type N-glycans, high-mannose-type N-glycans)	0
52	OVM	Ovomucoid (Complex-type N-glycans)	0
53	OVA	Ovoalbumin (Hybrid-type N-glycans)	0

54	aMan	Mana1-PAA	0
55	bMan	Manb1-PAA	0
56	[6P]Man	($6OPO_4$)Mana1-PAA	0
57	INV	Yeast invertase (High mannose-type N-glycans)	0
58	Tn	GalNAca1-PAA	0
59	Core1	Galb1-3GalNAca1-PAA	11200
60	Core2	Galb1-3(GlcNAcb1-6)GalNAca1-PAA	19700
61	Core3	GlcNAcb1-3GalNAca1-PAA	0
62	Core4	GlcNAcb1-3(GlcNAcb1-6)GalNAca1-PAA	0
63	Forssman	GalNAca1-3GalNAcb1-PAA	0
64	Core6	GlcNAcb1-6GalNAca1-PAA	0
65	Core8	Gala1-3GalNAca1-PAA	0
66	[3'S]Core1	($3OSO_3$)Galb1-3GalNAca1-PAA	0
67	Galb-Core3	Galb1-4GlcNAcb1-3GalNAca1-PAA	0
68	Asialo-BSM	Asialo bovine submaxillary mucin (Tn)	0
69	Asialo-GP	Asialo human glycophorin MN (T)	300
70	STn	Neu5Aca2-6GalNAca1-PAA	0
71	STn (Gc)	Neu5Gca2-6GalNAca1-PAA	0
72	ST	Neu5Aca2-3Galb1-3GalNAca1-PAA	0
73	Siaa2-6Core 1	Galb1-3(Neu5Aca2-6)GalNAca1-PAA	6100
74	BSM	Bovine submaxillary mucin (Sialyl Tn)	0
75	GP	Human glycophorin (Disialyl T and sialyl Tn)	0
76	aGal	Gala1-PAA	0
77	Gala1-2Gal	Gala1-2Galb1-PAA	0
78	Gala1-3Gal	Gala1-3Galb1-PAA	0
79	Gala1-3Lac	Gala1-3Galb1-4Glcb1-PAA	0
80	Gala1-3LN	Gala1-3Galb1-4GlcNAcb1-PAA	0
81	Gala1-4LN	Gala1-4Galb1-4GlcNAcb1-PAA	0
82	Melibiose	Gala1-6Glcb1-PAA	0
83	aGlc	Glca1-PAA	0
84	bGlc	Glcb1-PAA	0
85	Maltose	Glca1-4Glcb1-PAA	0
86	HA	Hyaluronic acid-BSA	0
87	CSA	Chondoroitin Sulfate A-BSA	0
88	CSB	Chondoroitin Sulfate B-BSA	0
89	HS	Heparan Sulfate-BSA	0
90	HP	Heparin-BSA	200
91	KS	Keratan Sulfate-BSA	0
92	aRha	Rhamnosea1-PAA	0
93	Mannan (SC)	*S. cerevisiae* mannan	0
94	Mannan (CA)	*C.albicans* mannan	0
95	Zymosan	Zymosan	0
96	Chitobiose	GlcNAcb1-4GlcNAcb1-PAA	0
97	BSA	-	0
98	Negative PAA	-	0
99	Marker		
100	BG		

Supplementary Table S2

List of proteins detected by mass spectrometry from 5G2 immunoprecipitated samples along with cDNA microarray data.

	Accession	Gene Name	5G2 C45 ΣSCORE	MS fold change C45/HCT116	MA C45/HT29
1	P55011	SLC12A2	4824.17	S	0.43
2	Q9H3R2	MUC13	4513.01	S	11.70
3	Q9Y666	SLC12A7	1984.21	S	1.27
4	Q92673	SORL1	1586.54	S	1.23
5	Q7Z7M9	GALNT5	1538.72	S	75.35
6	Q7Z3C6	ATG9A	1432.61	S	0.90
7	P08195	SLC3A2	1161.39	S	1.79
8	P02786	TFRC	1008.99	8.42	2.08
9	P16615	ATP2A2	992.95	S	0.72
10	P06731	CEACAM5	951.62	9.90	190.77
11	Q15758	SLC1A5	931.14	S	0.68
12	Q9UIQ6	LNPEP	902.56	S	1.06
13	O15438	ABCC3	897.32	S	1.00
14	P05026	ATP1B1	893.74	S	1.78
15	P17301	ITGA2	868.72	S	2.08
16	Q93050	ATP6V0A1	790.46	57.78	2.12
17	Q9H7F0	ATP13A3	718.08	S	1.63
18	Q63HN8	RNF213	711.79	S	1.68
19	P27487	DPP4	704.81	S	3.68
20	Q9BZC7	ABCA2	646.07	S	0.82
21	O43493	TGOLN2	622.67	S	1.00
22	P33527	ABCC1	617.34	S	1.85
23	O75976	CPD	614.54	S	0.50
24	P04920	SLC4A2	603.38	S	0.64
25	P30825	SLC7A1	603.22	S	0.59
26	Q9H8J5	MANSC1	588.30	S	1.45
27	Q8IZA0	KIAA0319L	566.73	S	0.73
28	P40199	CEACAM6	557.64	S	58.50
29	P05556	ITGB1	552.38	S	0.78
30	O00461	GOLIM4	492.76	S	4.27
31	P08962	CD63	489.26	S	1.28
32	Q92508	FAM38A	461.89	S	0.82
33	P13688	CEACAM1	459.48	S	3.09
34	Q8TD43	TRPM4	438.93	S	1.23
35	Q9BQS7	HEPH	425.34	S	22.48

5G2 C45 Σscore: total score of each proteins detected by mass spectrometry in immunoprecipitated samples with 5G2 mAb from floating cultured C45 CTOSs. MS fold change C45/HCT116: the ratio of total score of immunprecipitated samples with 5G2 mAb from C45 to that from HCT116. S indicates proteins detected specifically in C45. MA C45/HT29: the ratio of expression levels of mRNA coding each protein in floating cultured C45 to HT29 cells analyzed by cDNA microarray analysis.
